# Tuning optical absorption in perovskite (K,Na)NbO_3_ ferroelectrics†

**DOI:** 10.1039/d4ma00396a

**Published:** 2024-10-15

**Authors:** V. Vetokhina, N. Nepomniashchaia, E. de Prado, O. Pacherova, T. Kocourek, S. S. Anandakrishnan, Y. Bai, A. Dejneka, M. Tyunina

**Affiliations:** a Institute of Physics of the Czech Academy of Sciences Na Slovance 2 18221 Prague Czech Republic; b Microelectronics Research Unit, Faculty of Information Technology and Electrical Engineering, University of Oulu P. O. Box 4500 FI-90014 Oulu Finland marina.tjunina@oulu.fi

## Abstract

The ability to tailor the electronic band structure and optical absorption by appropriate cationic substitution in perovskite oxide ferroelectrics is essential for many advanced electronic and optoelectronic applications of these materials. Here, we explored weak (Ba,Ni)-doping for reducing optical bandgaps in (K,Na)NbO_3_ ferroelectric films and ceramics. The optical absorption in the broad spectral range of (0.7–8.8) eV was investigated in polycrystalline doped, pure, and oxygen deficient films, in doped epitaxial films grown on different substrates, and in doped ceramics. By comparing optical properties of all films and ceramics, it was established that 1–2 at% of cationic substitutions or up to 10 at % of oxygen vacancies have no detectable effect on the direct (∼4.5 eV) and indirect (∼3.9 eV) gaps. Concurrently, substantial sub-gap absorption was revealed and ascribed to structural band tailing in epitaxial films and ceramics. It was suggested that owing to fundamental strain-property couplings in perovskite oxide ferroelectrics, inhomogeneities of lattice strain can lead to increased sub-gap absorption. The uncovered structurally induced sub-gap optical absorption can be relevant for other ferroelectric ceramics and thin films as well as for related perovskite oxides.

## Abstract 2

Inhomogeneities of lattice strain can lead to substantial sub-gap optical absorption in perovskite oxide ferroelectrics.

## Graphical abstract

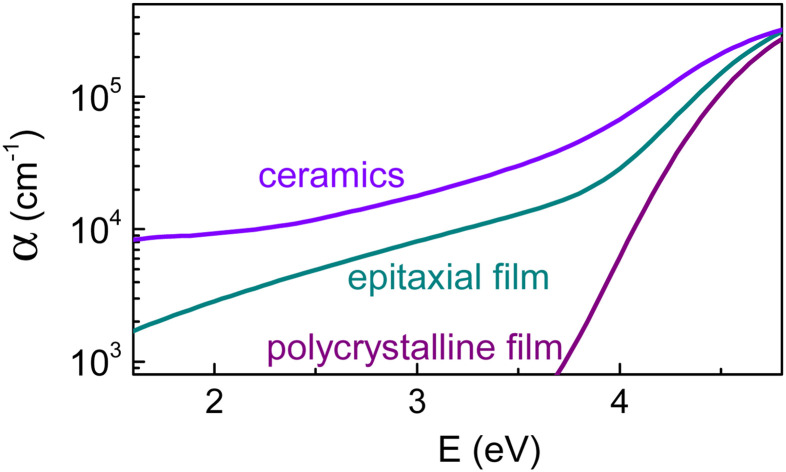

## Introduction

Excellent dielectric, piezoelectric, and electrooptic properties of ABO_3_ perovskite-type ferroelectrics (FEs) enable mainstream applications of these materials in diverse capacitors, electromechanical devices, and photonics.^1–3^ These applications also benefit from the fundamental electronic properties including wide bandgaps (>3 eV) and insulating behaviour of FEs.^1^ However, modern advanced FE applications in photovoltaics, detectors, resistive switching, and others require significant modifications of the electronic properties.^4–8^ Namely, for solar-reliant applications, it is desirable to raise the optical absorption in the visible spectral range (<3 eV). Being proven successful for tuning miscellaneous characteristics of FEs, cationic (and/or anionic) substitutions are expected to be efficient for tailoring the electronic properties as well.

Primarily, substitutions can lead to the formation of additional levels in band structure. For appropriate dopants, which create rather shallow in-gap levels (*i.e.*, those close to the conduction band minima or valence band maxima), one may anticipate an apparent bandgap narrowing. To achieve a measurable optical bandgap narrowing, the concentration of the dopants should be sufficiently high. Also, the narrowing is limited by the levels’ in-gap depth. On the other hand, if dopants induce deep in-gap levels, one may foresee additional absorption peaks in the spectral range of transparency of the original pure material. Importantly, the rate of direct band-to-band transitions is orders of magnitude larger than that for indirect and dopant-induced ones in perovskite oxide ferroelectrics. Based on these simplified fundamental considerations, it is difficult to expect that weak cationic doping can easily lead to significant optical absorption coefficient, whose magnitude is of the same order as for direct band-to-band transitions, or, in other words, to an assessable optical direct bandgap narrowing. Nevertheless, experimental observations of the dramatically reduced direct optical bandgaps have been reported for weakly doped (∼2 at%) FE ceramics.^9–13^ These controversial extraordinary observations imply that the explicit role of cationic substitutions in the optical absorption of FEs is not fully apprehended. It is worth noting that substitutions may also induce changes in crystal structure,^13^ which, in turn, may affect the electronic band structure and the optical absorption.

In this work, to better elaborate tuning of the optical bandgaps by cationic substitutions in FEs, we focused on the optical absorption in perovskite FE (K_0.5_Na_0.5_)NbO_3_ (or KNN) weakly doped with (Ba,Ni) (or BN-KNN for brevity). To disentangle effects of doping and microstructure, we examined ceramics and thin films. The chemical composition and crystal structure of the films were varied. To ensure assessment of the optical properties in opaque ceramics and in thin films on thick substrates, we applied reflection spectroscopic ellipsometry. Based on the analysis of the spectra and magnitude of the absorption coefficient, we detected structurally induced band tailing, whereas the gaps were unaffected by doping. We suggest that strain inhomogeneities can lead to band tailing and thus raise sub-gap absorption in perovskite oxide ferroelectrics.

## Experimental

Ceramics were prepared by solid state reaction as described before.^14,15^ The starting powder reactants BaCO_3_ (99.98%), Nb_2_O_5_, (99.9%), and NiO (99.999%) (Aldrich Chemistry, USA), K_2_CO_3_ (>99%) (J.T. Baker, USA), and Na_2_CO_3_ (>99%) (Sigma-Aldrich, USA) were ball-milled and calcined at 825 °C. The calcined and ball-milled powder was pressed into pellets (uniaxial pressure of 90 MPa). The ceramics were sintered at 1150 °C in air ambience. The sintered ceramic pellets were polished for use as targets in pulsed laser deposition and optically polished for analysis by spectroscopic ellipsometry.

Thin films of doped BN-KNN and pure KNN were grown by pulsed laser deposition using an excimer KrF laser. The key deposition parameters were as follows: laser fluence 2 J cm^−2^, substrate temperature 700 °C, oxygen pressure 20 Pa for regular films and 0.1 Pa for oxygen-deficient films. The lowered oxygen pressure of 0.1 Pa resulted in ∼10 at% oxygen vacancies.^16–19^ Single-crystal (001)-oriented (LaAlO_3_)_0.3_(Sr_2_AlTaO_6_)_0.7_ (LSAT) and SrTiO_3_ (STO) substrates (MTI. Corp., USA) were employed to enable epitaxial growth, whereas SiO_2_-coated Si and silica substrates were used for deposition of polycrystalline films. The nominal thickness of the films was regulated by the number of laser pulses and varied from ∼50 nm to ∼150 nm.

The elemental composition of all the samples was characterized by X-ray photoelectron spectroscopy (XPS) on an ESCALAB 250Xi XPS instrument (Thermo Fisher Scientific) and by X-ray fluorescence spectroscopy (XRF) on an Orbis PC Micro-XRF spectrometer (EDAX, Ametek). Crystal structure of the films was inspected by X-ray diffraction (XRD) using Cu Kα radiation on a SmartLab SE Multipurpose diffractometer (Rigaku Corp.), a D8 DISCOVER diffractometer (Bruker Corp.), and an Empyrean diffractometer (Malvern Panalytical). Also X-ray reflectivity (XRR) was measured on the D8 DISCOVER diffractometer.

The optical properties of the films, substrates, and optically polished ceramics were studied using variable angle spectroscopic ellipsometry. The measurements were performed on a J. A. Woollam VUV VASE ellipsometer at room temperature. The optical dielectric functions were extracted from the spectra of ellipsometric angles using a commercial WVASE32 software package (ESI,† Fig. S1). The optical absorption coefficient was calculated from these dielectric functions. More details on the measurements and data processing can be found in our previous works.^20–22^

## Results and discussion

**Fig. 1:**
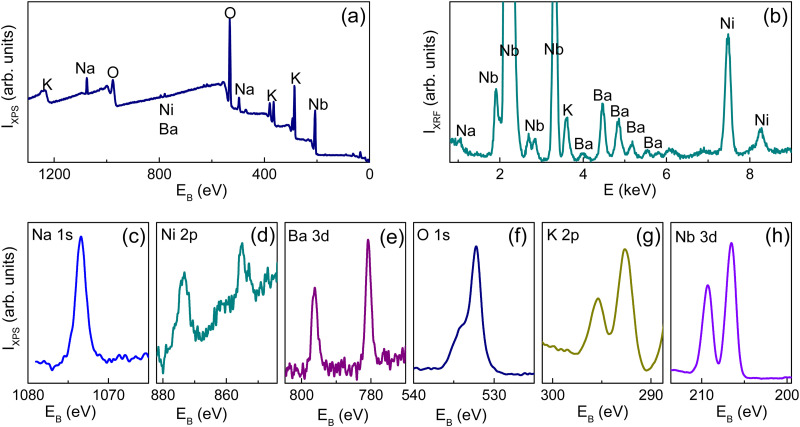
Typical (a) XPS survey spectrum and (b) XRF spectrum. The characteristic lines of K, Na, Nb, Ba, Ni, and O are marked on the plots. (c)–(h) Details of the selected characteristic XPS lines.

The performed XPS and XRF analyses evidenced the presence of Ba and Ni dopants in BN-KNN (Fig. 1). The chemical composition was quantified using a combination of both the XPS spectra containing strong lines of K, Na, Nb, and O, and XRF spectra containing strong lines of Nb, Ba, and Ni. The content of Nb was considered as a reference to match the data. Assuming ABO_3_-type perovskite formula unit with the A-site Ba and B-site Ni substitutions, the estimated composition of BN-KNN can be described as (K_0.5_Na_0.5_)_0.98_Ba_0.02_Nb_0.99_Ni_0.01_O_3_. Discernible compositional variations in different BN-KNN samples were not detected.

**Fig. 2:**
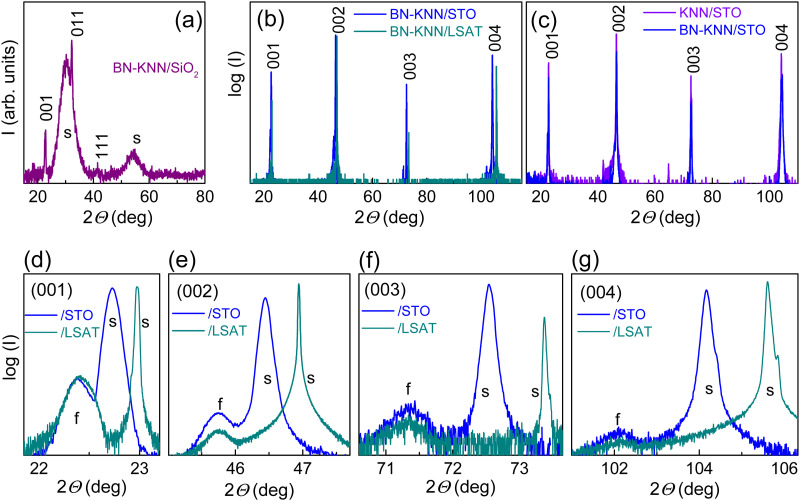
High resolution XRD (a) grazing incidence and (b)–(g) *ω*−2*Θ* scans in the films on (a) SiO_2_/Si and (b)–(g) STO and LSAT substrates. In (c), the pure KNN film on STO is shown for comparison. In (a)–(c), perovskite peaks are indexed. In (d)–(g), details of the scans around the (d) (001), (e) (002), (f) (003), and (g) (004) peaks are shown. In (a) and (d)–(g), the peaks from the films and substrates are marked by “f” and “s”, correspondingly.

The structural XRD analysis affirmed the formation of perovskite phase in the films (Fig. 2). Randomly oriented polycrystalline films grew on Si and SiO_2_ substrates (Fig. 2a). The room-temperature crystal structure of the films on STO and LSAT can be interpreted as pseudo-cubic perovskite, oriented, with the (00*l*) planes parallel to those of the substrates (Fig. 2b). Effects of the Ba and Ni dopants were not detected in the films (Fig. 2c). The measured average out-of-plane lattice parameters (∼0.396 nm) were similar for the films on STO and LSAT (Fig. 2d–g). The parameters implied that the large compressive in-plane strain induced by film-substrate lattice mismatch (approximately −2% for STO and −3% for LSAT at room temperature) was relaxed.

For the film-substrate lattice misfit of 2–3%, the critical thickness for strain relaxation is small, <10 nm.^23^ The relaxation of strain leads to a distribution of strain across the thickness of the film and to local fluctuations of strain.^19,23,24^ The resultant strain inhomogeneities determine complex shapes of XRD peaks,^19^ that is seen as a convoluted peak broadening in experimental XRD scans. It is likely that the larger film-substrate mismatch produces strain inhomogeneities of larger magnitude and wider distribution in the films on LSAT than on STO. Despite profound strain relaxation, the films on LSAT and STO were cube-on-cube-type epitaxial (ESI,† Fig. S2).

**Fig. 3:**
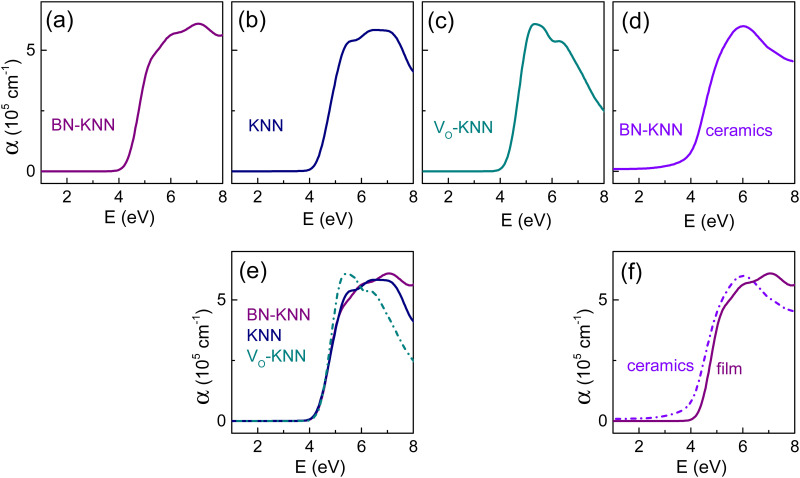
Absorption coefficient as a function of photon energy in the polycrystalline films of (a) doped BN-KNN, (b) pure KNN, (c) oxygen-deficient KNN, and (d) in doped BN-KNN ceramics. In (e), absorption coefficient in the films of different compositions is shown for ease of comparison. In (f), absorption coefficient in the polycrystalline BN-KNN film and ceramics is shown for ease of comparison.

**Fig. 4:**
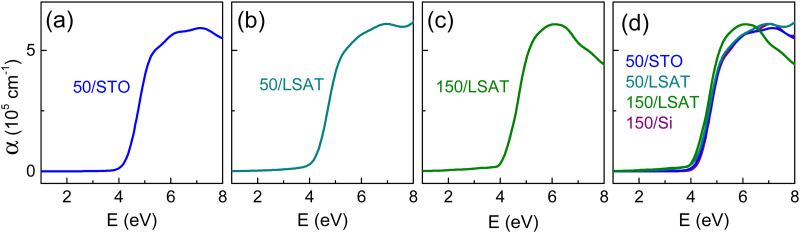
Absorption coefficient as a function of photon energy in the epitaxial BN-KNN films on (a) STO and (b) and (c) LSAT substrates. The nominal films thickness is (a, d) and (b, d) 50 nm and (c, d) 150 nm. In (d), absorption coefficient in the BN-KNN epitaxial and polycrystalline films is shown for ease of comparison.

The coefficient of optical absorption α was calculated from the dielectric functions obtained by spectroscopic ellipsometry in the polycrystalline films of different compositions, BN-KNN ceramics, and BN-KNN epitaxial films on different substrates (Fig. 3 and 4). Inspections of the 150 nm thick polycrystalline films of doped BN-KNN, pure KNN, and oxygen-deficient V_O_-KNN (V_O_ denotes oxygen vacancy) showed that the fundamental absorption sets at photon energy of ∼4 eV independently of composition (Fig. 3a–c). The films’ absorption spectra practically coincide in the range up to ∼5 eV (Fig. 3e).

The coefficient α steeply increases at approximately 4 eV also in BN-KNN ceramics (Fig. 3d), similarly to that in polycrystalline films. However, compared to the polycrystalline BN-KNN film, the edge of absorption is slightly red-shifted in ceramics. Furthermore, the coefficient α is noticeably larger in ceramics for the photon energy below 4 eV.

The absorption spectra in Fig. 3 indicate that the cationic substitutions and/or oxygen vacancies have negligible, if any, effect on the absorption edge (Fig. 3e), whereas the edge may be influenced by microstructural factors (Fig. 3f).

Inspections of different structurally relaxed epitaxial BN-KNN films revealed minor variations of the edge (Fig. 4). Compared to polycrystalline films, the absorption coefficient was found to be somewhat higher in the spectral range below 4 eV (Fig. 4) in epitaxial films. These observations (Fig. 4d) also point to the possible role of microstructure in the absorption edge.

**Fig. 5:**
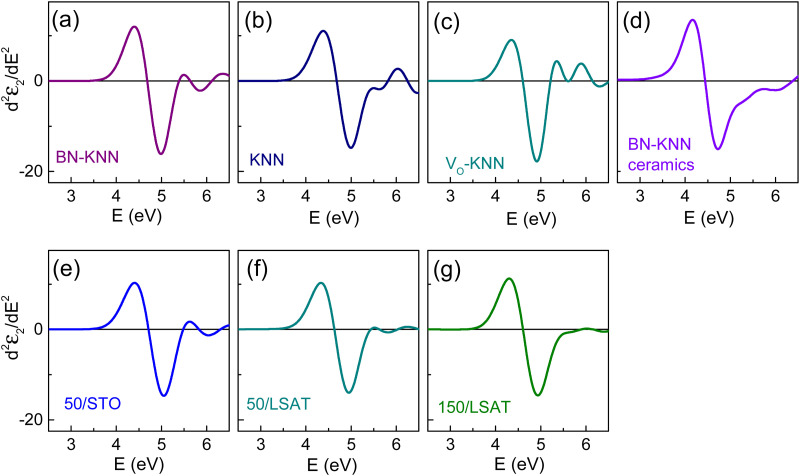
Second derivative of the imaginary part of the dielectric function as a function of photon energy in the (a)–(c) polycrystalline films of (a) doped BN-KNN, (b) pure KNN, (c) oxygen-deficient KNN, (d) BN-KNN ceramics, and (e)–(g) BN-KNN epitaxial films on (e) STO and (f) and (g) LSAT.

To get better insight into the electronic transitions responsible for the absorption edge, we next determined the gaps using the critical point (CP) analysis^22,25,26^ and Tauc plots.^27–32^ The spectra of the second derivative of the imaginary part of the dielectric function, *d*^2^*ε*_2_/d*E*^2^, unveiled the strongest CP with the energy ∼4.5 eV in all films and ceramics (Fig. 5). This CP corresponds to the lowest-energy direct band-to-band transition and is typical for perovskite oxide FEs.^20–22^ The CP energy *E*_CP_ was approximated as that for *d*^2^*ε*_2_/d*E*^2^ = 0. The obtained *E*_CP_ values are listed in Table 1.

**Table 1:** Energies of the main CP *E*_CP_, direct gap *E*_D_, indirect gap *E*_I_, and Urbach energy *E*_U_ and coefficient α_0_ in different films (thickness *D*_N_) and ceramics. The energies are determined with the accuracy of not worse than ±0.02 eV

Structure	Composition	*D*_N_, nm	*E*_CP_, eV	*E*_D_, eV	*E*_I_, eV	*E*_U_, eV	*a*_0_, 10^4^ cm^−1^
POLYCRYST	BN-KNN	150	4.68	4.63	3.93	—	—
	KNN	150	4.67	4.63	3.93	—	—
	V_O_-KNN	150	4.60	4.52	3.86	—	—
CERAMICS	BN-KNN	Bulk	4.45	4.43	—	1.06	6.9
EPITAX/STO	BN-KNN	50	4.70	4.61	3.88	0.56	1.3
EPITAX/LSAT	BN-KNN	50	4.62	4.61	3.86	1.05	3.5
EPITAX/LSAT	BN-KNN	150	4.60	4.53	3.63	1.07	4.5

**Fig. 6:**
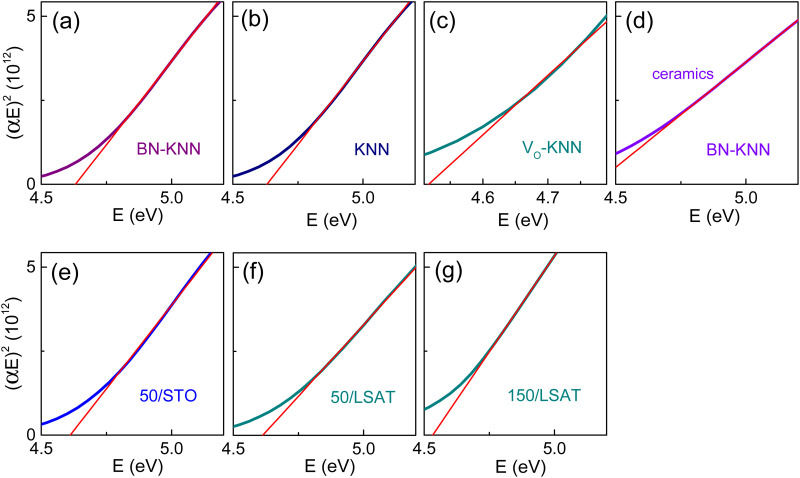
Tauc plots for direct optical gap in the (a)–(c) polycrystalline films of (a) doped BN-KNN, (b) pure KNN, and (c) oxygen-deficient KNN; in (d) BN-KNN ceramics, and (e)–(g) BN-KNN epitaxial films on (e) STO and (f) and (g) LSAT. Straight lines show fits.

The lowest-energy direct band-to-band transition determines the direct bandgap, whose energy *E*_D_ was also evaluated using linear fits to Tauc plots for direct gap [(*αE*)^2^ ∝ (*E* – *E*_*D*_)]. Good fits were gained in all films and ceramics (Fig. 6). The direct bandgap energies *E*_D_ determined from the fits agree well with the energies *E*_CP_ of the main CP (Table 1).

The results in Fig. 3–6 evidence approximately the same direct bandgaps in all studied samples. It is very unlikely that variations in the chemical composition and/or microstructure can lead to a significant (on the order of 1–2 eV) reduction of the direct gap. As is worth mentioning, the rate of direct interband transitions is fundamentally very high so that the absorption coefficient associated with the direct interband transitions is large ∼10^5^–10^6^ cm^−1^. Therefore, the Tauc plots for direct gaps were analysed in the spectral region of high absorption. The coefficient was *α* ≈ (3⋯5) × 10^5^ cm^−1^ for the fits in Fig. 6.

**Fig. 7:**
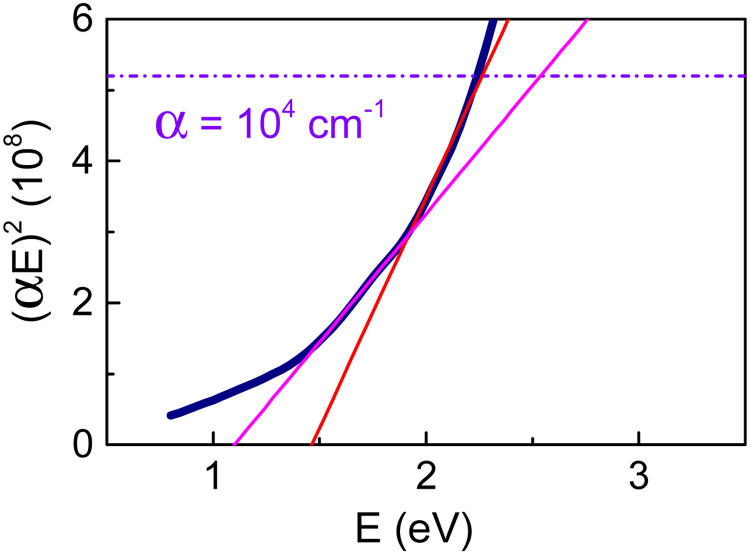
Formal Tauc plot for direct gap in the range of weak absorption in ceramics. Dashed line shows the level of *α* = 10^4^ cm^−1^. Straight lines are possible fits.

Formally applying Tauc plots for analysis of direct gaps in the region of weak absorption can be misleading. As an example of such a formal approach, we probed linear fits to Tauc plot for direct gap in the range of low absorption *α* < 10^4^ cm^−1^ in BN-KNN ceramics (Fig. 7). The correspondingly determined bandgap energies are artificially small *E*_D_ < 1.5 eV. We note that optical characterization of opaque FE ceramics is commonly performed by inspections of diffuse reflectance with Kubelka–Munk function, which does not provide the explicit magnitude of the absorption coefficient.^33–37^

**Fig. 8:**
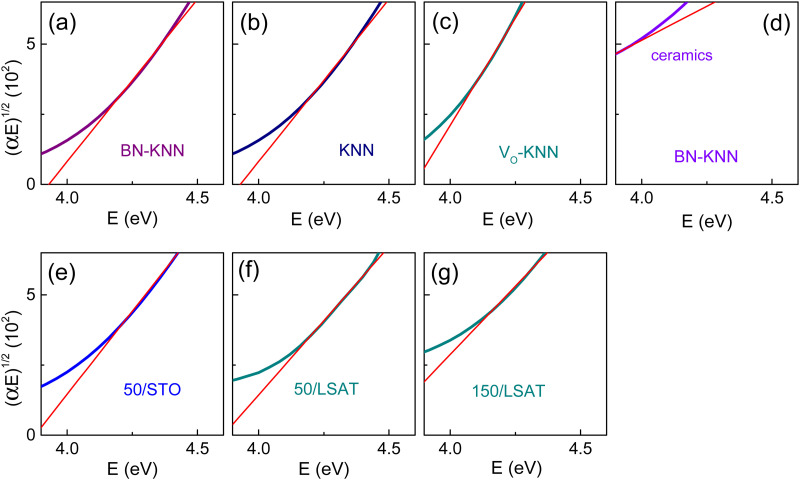
Tauc plots for indirect optical gap in the (a)–(c) polycrystalline films of (a) doped BN-KNN, (b) pure KNN, and (c) oxygen-deficient KNN; in (d) BN-KNN ceramics, and (e)–(g) BN-KNN epitaxial films on (e) STO and (f) and (g) LSAT. Straight lines show fits.

To evaluate the energy *E*_I_ of indirect bandgap, Tauc plots for indirect gap [(*αE*)^1/2^ ∞ (*E* – *E*_I_)] were analysed in the range of *α* < 7 × 10^4^ cm^−1^ in the vicinity of the absorption edge at ∼4 eV (Fig. 8). The linear fits were decent and gave the energy *E*_I_ ≈ 3.9 eV in the films (Table 1). The indirect bandgap energies affirm that weak cationic doping cannot significantly reduce bandgaps.

It appeared impossible to unambiguously identify indirect bandgap in ceramics, where the absorption coefficient was found to be high, *α* > 10^4^ cm^−1^, in the broad spectral range from as low photon energy as ∼2 eV up to ∼4 eV (ESI,† Fig. S3).

**Fig. 9:**
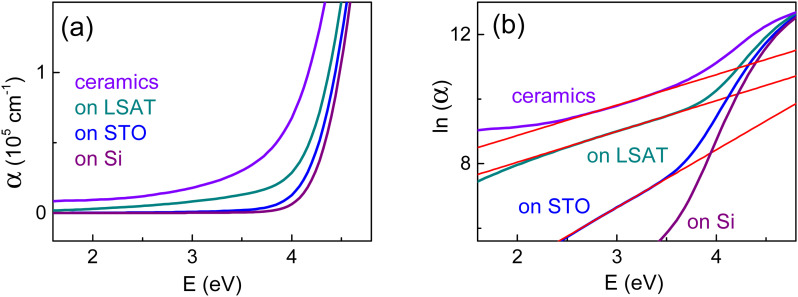
(a) Absorption coefficient and (b) logarithm of absorption coefficient ln(*α*) as a function of photon energy in different BN-KNN samples as marked on the plots. In (b), straight lines show fits.

We stress that high ceramics-type sub-gap absorption was not observed in polycrystalline films. Although, significant sub-gap absorption was detected in the epitaxial films on LSAT and was also seen in the epitaxial films on STO (Fig. 9a). The sub-gap absorption coefficient at photon energy of 3 eV was nearly 2 × 10^4^ cm^−1^ in ceramics and ∼1 × 10^4^ cm^−1^ in the films on LSAT. Such a high magnitude discards methodological origin and proves validity of the observed sub-gap absorption.

To closer examine the high sub-gap absorption, we considered a general Urbach-type absorption tail: *α* = *α*_0_ exp {(*E* − *E*_0_)/*E*_U_}.^38–46^ The Urbach energy *E*_U_ shows the in-gap depth of the band tailing. The absorption tails were below detection limit in the polycrystalline films. Good linear fits to [ln(*α*) ∝ *E*] were found in the sub-gap spectral range in the epitaxial films and ceramics (Fig. 9b). We emphasize that this Urbach-type behaviour is quite different from the Drude-type absorption caused by free carriers. The energy *E*_U_ was estimated from the fits and was found to reach ∼0.6 eV in the films on STO and ∼1 eV in the films on LSAT and in ceramics (Table 1). The accessed energy *E*_U_ is huge compared to the typical magnitude of a few tens millielectronvolts for thermally induced band tailing.^38,46^ The found large *E*_U_ signifies that the band tailing can be related to structural factors.

Because the tailing was found in epitaxial films, it could not be caused by structural disorder (amorphous phase).^38^ Importantly, randomly oriented polycrystalline films did not exhibit tailing. Thus, structural inhomogeneities like different crystal orientations and phase boundaries, could not be responsible for the tailing either.

Importantly, the Urbach energy *E*_U_ and coefficient *α*_0_ are found to be larger in the films on LSAT than on STO. These observations indicate that stronger tailing can be associated with weightier inhomogeneities of lattice strain. In epitaxial films, strain inhomogeneities originate from the relaxation of substrate-induced lattice misfit.^19,23,24^ The revealed stronger tailing in the relaxed films on LSAT than on STO is in line with more profound strain inhomogeneities (*i.e.*, larger magnitudes and wider distribution for local strains) resulting from the larger film-substrate mismatch on LSAT. The lack of pronounced sub-gap absorption in the polycrystalline films is coherent with the absence of such strain inhomogeneities therein.

Based on the observations in the polycrystalline and epitaxial films, we anticipate that strain inhomogeneities can be largely responsible for the exceptionally high sub-gap absorption in BN-KNN ceramics. This expectation is solidly supported by the previously reported correlation between sintering conditions and optical absorption for such ceramics.^10^

The main characteristics of the optical absorption in the studied films and ceramics are summarized in Table 1. These data evidence that the presence of weak cationic Ba and Ni doping or up to 10 at% of oxygen vacancies has negligible effect on the direct and indirect gaps in KNN. The Urbach-type sub-gap absorption tails are non-detectable in polycrystalline films, profound in epitaxial films, and massive in ceramics. The found large Urbach energy indicates structurally induced band tailing. The tailing is suggested to be related to inhomogeneities of lattice strain. Strong coupling between lattice strain and all properties of perovskite oxide FEs is well established.^1,47,48^ Likewise, FE properties can be modified by strain gradients.^49,50^ Here, we suggest that strain inhomogeneities, or the presence of nanoscale-sized regions with different local strains and/or strain gradients, can affect band edges on average. This suggestion is consistent with the fundamental effects of strain in FEs.^1,47–50^ Although explicit mechanism of strain-induced band tailing requires further investigations, we presume that strain inhomogeneities can lead to large sub-gap absorption in perovskite oxide FEs and other related materials.

## Conclusions

The possibility to control the optical bandgaps by weak (Ba,Ni) doping was explored in KNN ferroelectrics. The optical properties in the spectral range of (0.7–8.8) eV were investigated in thin polycrystalline doped BN-KNN, pure KNN, and oxygen deficient V_O_-KNN films, doped BN-KNN epitaxial films grown on different substrates, and doped BN-KNN ceramics. It was demonstrated that the presence of 1–2 at% of cationic substitutions or up to 10 at% of oxygen vacancies has little effect on the direct (∼4.5 eV) and indirect (∼3.9 eV) gaps in KNN. In the sub-gap spectral region, the absorption coefficient was negligibly small in all polycrystalline films, significant in epitaxial films, and massive ∼10^4^ cm^−1^ in ceramics. The sub-gap absorption was found to be Urbach-type and related to structural band tailing. It was suggested that owing to intrinsic strong strain-property couplings in perovskite oxide ferroelectrics, inhomogeneities of lattice strain can lead to band tailing. It was anticipated that structurally induced band tailing can raise sub-gap absorption in other ferroelectrics and related perovskite oxides.

## Author contributions

V. V.: investigation. N. N.: investigation. E. P.: investigation. O. P.: investigation. T. K.: investigation. S. S. A.: investigation. Y. B.: funding acquisition; supervision. A. D.: funding acquisition. M. T.: conceptualization; formal analysis; funding acquisition; supervision; writing – original draft; writing – review & editing.

## Data availability

The data supporting this article have been included as part of the ESI.†

## Conflicts of interest

There are no conflicts to declare.

## Supplementary Material

MA-005-D4MA00396A-s001
