# Supplementary material for: Tuning optical absorption in perovskite (K,Na)NbO3 ferroelectrics
Source: Mater Adv. 2024 Oct 15;5(22):8901–8. doi: 10.1039/d4ma00396a (PMC11492214; doi:10.1039/d4ma00396a)
Supplement: MA-005-D4MA00396A-s001 [file MA-005-D4MA00396A-s001.pdf]

Supplementary Information (SI) for Materials Advances.
This journal is © The Royal Society of Chemistry 2024

SUPPLEMENTARY INFORMATION

TO

Tuning optical absorption in perovskite (K,Na)NbO_3 ferroelectrics

V. Vetokhina,[1] N. Nepomniashchaia,[1] E. de Prado,[1] O. Pacherova,[1] T. Kocourek,[1] S. S. Anandakrishnan,[2] Y. Bai,[2] A. Dejneka,[1] M. Tyunina[1,2*]

[1]*Institute of Physics of the Czech Academy of Sciences, Na Slovance 2, 18221 Prague, Czech Republic*

[2]*Microelectronics Research Unit, Faculty of Information Technology and Electrical Engineering, University of Oulu, P. O. Box 4500, FI-90014 Oulu, Finland*

Spectroscopic ellipsometry

The dielectric function was presented by a combination of terms (oscillators)

$$\varepsilon = \frac{A_1}{En_1^2 - E^2} + \sum_{j=2}^{8} Gaussian(E, A_j, Br_j, En_j), \tag{S1}$$

where $Gaussian(E, A_j, Br_j, En_j)$ is a complex function with the following imaginary part:

$$Im(Gaussian(E, A_j, Br_j, En_j)) = A_j \times exp\left[-\left(\frac{E - En_j}{Br_j}\right)^2\right] - A_j \times exp\left[-\left(\frac{E + En_j}{Br_j}\right)^2\right]. \tag{S2}$$

Here *A* is the amplitude, *En* is the energy, and *Br* is the width of the oscillator, and *E* is the photon energy. By varying oscillator parameters, the dielectric function was established so that the difference between the correspondingly modelled ellipsometric data and the experimentally measured spectra was minimized (Fig. S1). Optical absorption coefficient α was calculated from the dielectric function

$$\alpha = \frac{4\pi E}{hc} \sqrt{0.5\left((\varepsilon_1^2 + \varepsilon_2^2)^{1/2} - \varepsilon_1\right)} \ , \tag{S3}$$

where *h* is the Planck's constant, *c* is the speed of light in vacuum, and ε_1 and ε_2 are the real and imaginary parts of the dielectric function.

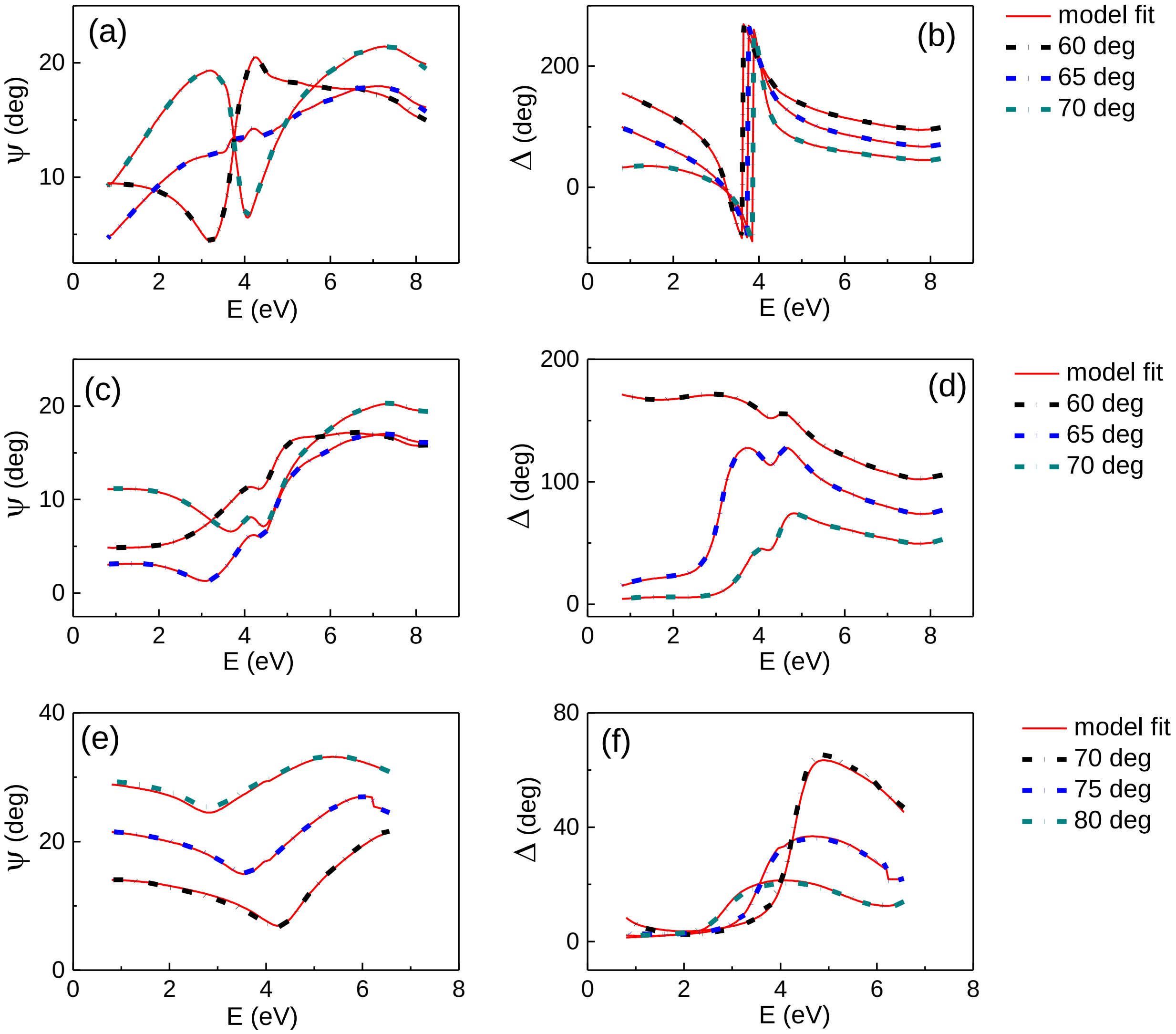

Fig. S1. (a-d) Ellipsometric angles (a,c,e) ψ and (b,d,f) Δ as a function of photon energy in BN-KNN films on (a, b) STO and (c, d) LSAT substrates and (e, f) BN-KNN ceramics. Dashed curves show experimental data acquired at different angles of incidence (55, 60, 65, 70, 75, and 80 deg, correspondingly). Solid red curves show fits to multi-oscillator models.

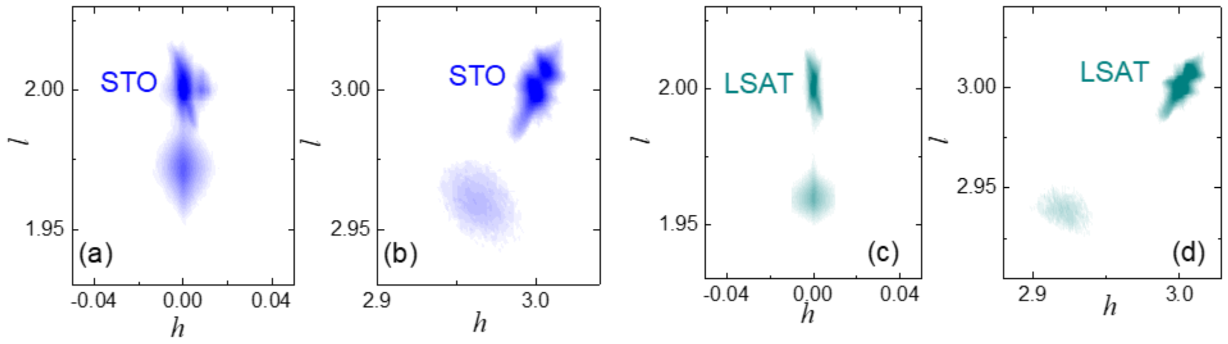

Fig. S2. Reciprocal space maps around (a, c) (002) and (b, d) (303) lattice points for BN-KNN films on (a, b) STO and (c, d) LSAT. Coordinates are expressed in reciprocal lattice units of substrates.

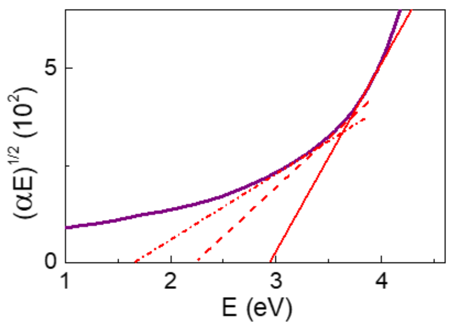

Fig. S3. Uncertainty of determining indirect gap in ceramics. Solid curve shows Tauc plot. Straight lines illustrate different formal fits in various very narrow spectral regions.
